# Assessing the Effectiveness and Accessibility of Trauma Care in East London: A Comprehensive Evaluation

**DOI:** 10.7759/cureus.84141

**Published:** 2025-05-15

**Authors:** Muhammed Monjur Ahmed, Ayman El-Zanaty, Mohammed Tanvir Shah, Shehryaar Kiani, Muhammad Subed Ali

**Affiliations:** 1 Trauma and Orthopaedics, Royal London Hospital, London, GBR; 2 Trauma and Orthopaedics, Royal National Orthopaedic Hospital, London, GBR; 3 Trauma and Orthopaedics, Southend University Hospital, London, GBR; 4 Trauma and Orthopaedics, Aintree University Hospital, Liverpool, GBR; 5 Trauma and Orthopaedics, Basildon University Hospital, London, GBR

**Keywords:** emergency service, health policy and management, london major trauma system, major trauma, major trauma centre, pre-hospital, public health care, trauma systems

## Abstract

East London has a distinct landscape for trauma care, balancing innovation and significant socioeconomic challenges. The Barts Health NHS Trust, found in East London, trauma care is critically examined in this evaluation, considering its successes in pre-hospital response, hospital care, rehabilitation, and prevention. Surrounding problems, such as community violence, budget shortages, and vulnerabilities in the data infrastructure, all impact trauma provision in this area. Looking ahead, to guarantee fair and efficient trauma care throughout the region, sustained improvements will necessitate concerted investment, legal change, and increased community involvement.

This is a structured literature review and policy analysis incorporating official local and regional government statistics, NHS data, government reports, peer-reviewed studies, and clinical outcomes between 2010 and 2024.

## Introduction and background

This paper aims to critically discuss the trauma provision in the East London region, outline strengths and weaknesses that may affect patient outcomes, and identify priority areas for improving trauma services in East London.

Outline of hospitals in East London

The Barts Health NHS Trust comprises four hospitals in the East London region: The Royal London Hospital, Newham General Hospital, Whipps Cross Hospital, and St Bartholomew’s Hospital. The Royal London Hospital is one of four major trauma centers in London and is a well-recognized institution within the Trust.

**Figure 1:**
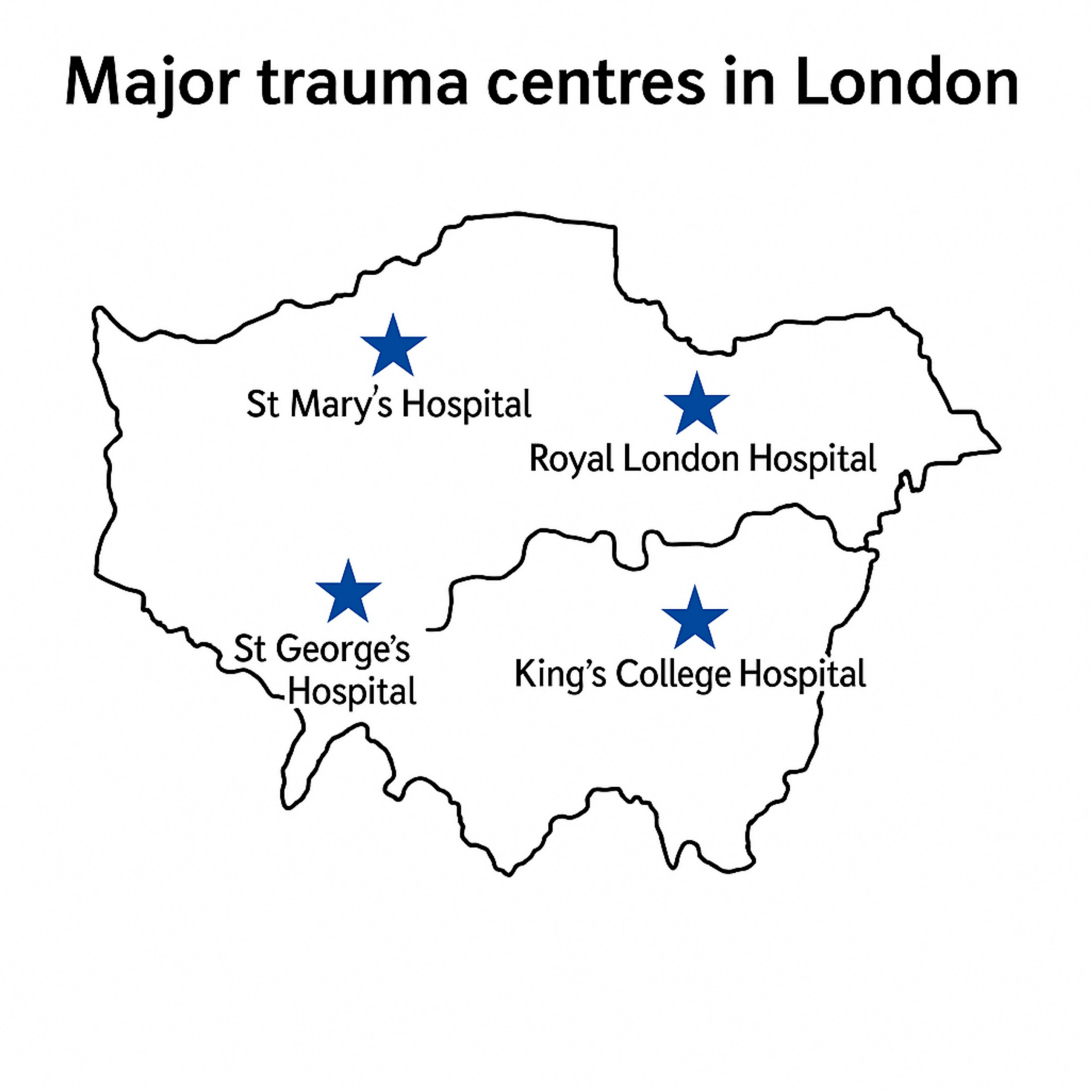
Major trauma centers in London. Image credits: Muhammed Monjur Ahmed.

Royal London Hospital is situated approximately one mile from Canary Wharf, one of the most prominent financial districts in the world, and one mile from Newham, one of the most socioeconomically deprived boroughs among London’s 32 boroughs [1]. Newham’s high population density [2] contributes to increased human interactions and a correspondingly high incidence of trauma. This dense population highlights the importance of a robust trauma system and presents significant challenges to trauma provision in the region (Figure 1).

What is a trauma system?

**Figure 2:**
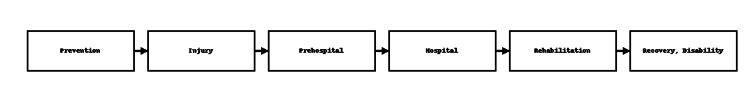
The diagram indicates the key steps of a patient’s timeline of injury and for a hospital to be inclusive it must aim to improve each stage of the patient’s plight. Image credits: Muhammed Monjur Ahmed.

The Barts Health NHS Trust is integral to the London Major Trauma System, which was the first organized trauma system in the United Kingdom, established in 2010 [3]. A trauma system is a structured organization of resources and services designed to provide comprehensive care to injured patients, from the moment of injury through to rehabilitation (Figure 2).

## Review

Strengths of trauma provision

Prevention Measures

London is one of the most densely populated cities in the Western world, with approximately 5,700 residents per square kilometer (as of 2021), and this figure continues to grow [2]. The frequency of trauma is therefore likely to increase from the current 10,000 trauma patients seen in London each year [3], particularly in the form of violence-related injuries and road traffic accidents (RTAs) [3], as greater numbers of people encounter one another.

The introduction and success of *Boris Bikes* in 2010 [3] have brought more cyclists onto the roads. Consequently, one in three trauma deaths is now caused by RTAs [3], making efforts to reduce road traffic collisions increasingly important. East London has been proactive in implementing a range of initiatives aimed at reducing trauma in the area. The introduction of dedicated bike lanes has provided an effective alternative method of travel, particularly given London’s busy rush hour periods and rising Transport for London (TfL) prices.

Referring to Newham’s road safety strategy [4-5], the installation of 20 mph zones and initiatives to improve traffic efficiency on major roads are likely to contribute to a reduction in cycling-related injuries. However, conducting an audit would be beneficial for the government and could support the expansion of these initiatives across the city. Additionally, the installation of more speed humps and the enforcement of increased speeding fines [6] have proven effective in deterring reckless driving, which is a common cause of presentations to the Emergency Department (ED).

Overall, encouraging progress has been made by the local government to reduce trauma morbidity, and preventative measures remain a key factor in maintaining effective trauma provision in East London.

Evolving Prehospital Care

According to local media, East London faces significant traffic congestion, making it extremely challenging for injured patients to receive prompt medical aid. Consequently, London’s Air Ambulance (formerly known as London Helicopter Emergency Services) has been pivotal in the care of major trauma patients. London’s Air Ambulance operates tirelessly, undertaking approximately five missions per day [3].

**Figure 3:**
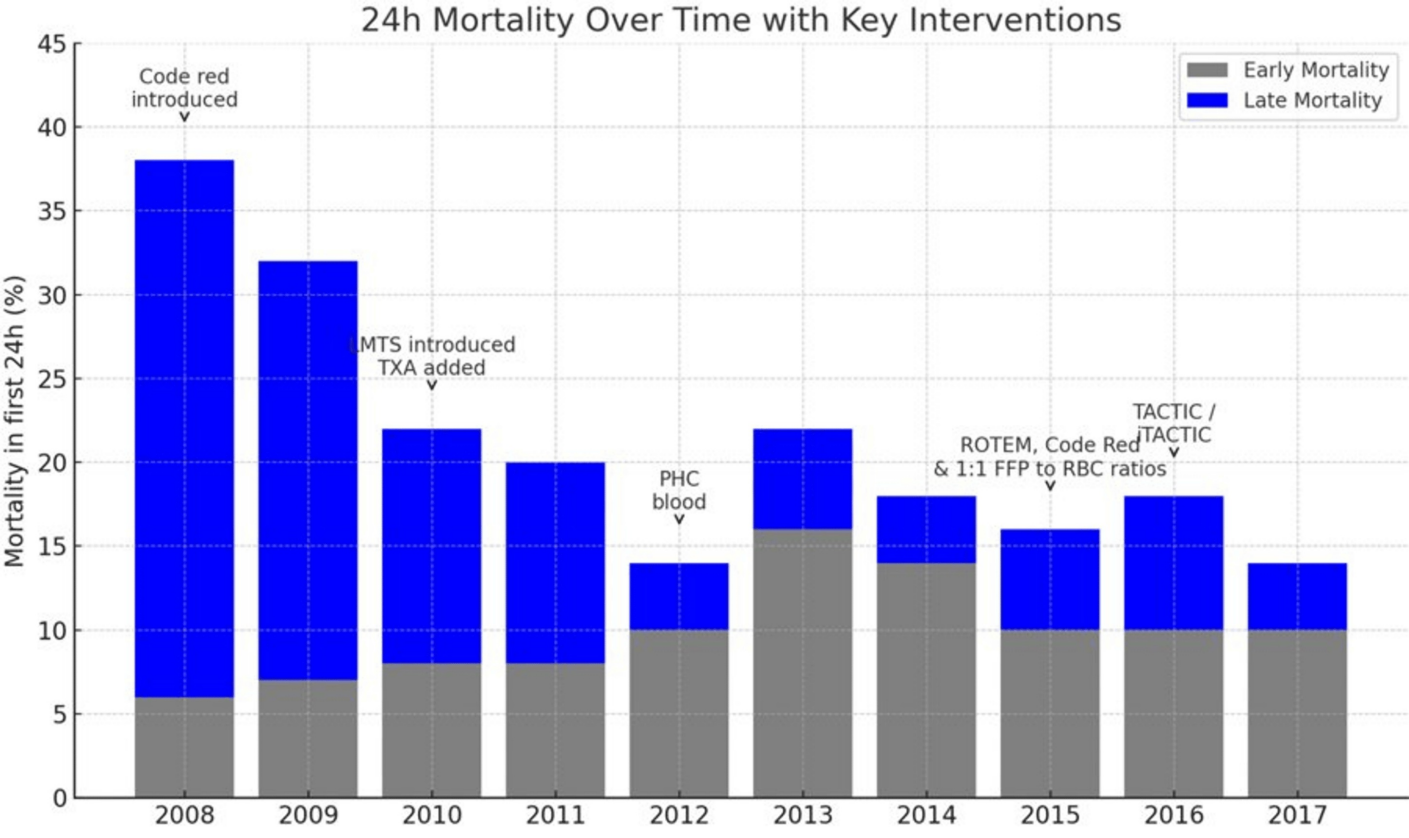
Twenty-four-hour mortality (%) in trauma patients from 2008 to 2017, with key interventions annotated. Notable interventions include the implementation of Code Red protocols, the introduction of tranexamic acid (TXA), prehospital blood (PHC) administration, and ROTEM-guided transfusion strategies. Image Credits: Muhammed Monjur Ahmed. ROTEM, Rotational Thromboelastometry; MTS, major trauma system

The crew configuration typically includes a consultant-level doctor, a paramedic, two pilots, and two fire crew members, delivering a wealth of knowledge and experience to manage critically unwell patients as early as possible, thereby improving their chances of survival. The service continues to evolve, with increasing numbers of case reviews and the adoption of innovative practices - for example, the introduction of carrying blood products on board in 2012, as shown in Figure 3.

The principle that *time is life* underpins trauma care, and any method that expedites patient transfer to the hospital has a significant impact on improving outcomes.

Advancements in Hospital Care

The Royal London Hospital has been at the forefront of trauma innovation. A notable example is the introduction of the *Damage Control Resuscitation* (DCR) strategy [7] and the *Major Haemorrhage Protocol *(MHP), also known as *Code Red* [8], both of which were adopted as national standards by the National Health Service (NHS) and the National Institute for Health and Care Excellence (NICE) in 2016 [9].

Professor Karim Brohi, a vascular surgeon at Barts Health NHS Trust, conducted groundbreaking research revealing that a substantial proportion of trauma patients exhibit acute traumatic coagulopathy (ATC) upon arrival at the ED - a condition that, in 2010, was associated with a critical bleeding mortality rate of 38% within the first twenty-four hours of injury [3]. In response, through robust clinical governance, the team introduced the DCR protocol, which reduced mortality from exsanguination by 25% between 2010 and 2020 at the Royal London Hospital [3].

In addition to the clinical impact, from a financial and resource perspective, the NHS achieved savings of £6.6 million annually, and it was predicted that adequate social cost savings over five years would amount to £3.6 billion (based on 2010 prices), largely due to the reduction in red cell transfusion use. Instead, a fixed ratio of blood products was incorporated into resuscitation strategies. Following the success of this research, the NHS implemented specific MHPs for both adults and children and introduced the use of tranexamic acid (TXA) to treat acute coagulopathy in trauma patients on arrival [9].

These changes have significantly impacted mortality rates from 2008 to 2017, as highlighted in Figure 3, and have been incorporated into the UK national transfusion guidelines for major bleeding. They have also influenced the European guidelines for the management of trauma hemorrhage, the global Advanced Trauma Life Support (ATLS) manual, guidelines from the British Society of Haematology, and the recommendations of the International Viscoelastic Testing in Trauma Consensus Panel.

Importance of Rehabilitation

Rehabilitation is defined as a process of assessment, treatment, and management, with ongoing evaluation, through which the individual (and their family or carers) is supported to achieve their maximum potential for physical, cognitive, social, and psychological function, participation in society, and quality of life [10].

Barts Health NHS Trust recognizes that rehabilitation is critical for improving long-term patient outcomes. Accordingly, the Royal London Hospital has established an after-trauma care team comprising physiotherapists, occupational therapists, speech and language therapists (SALT), psychologists, and physiatrists. This multidisciplinary expertise enhances patient mobility, promotes self-management, reduces the rate of reinjury, and supports reintegration into society.

Furthermore, research [11] highlights a significant rate of reinjury associated with violence-related trauma. Consequently, the introduction of safeguarding measures and comprehensive after-trauma care initiatives may reduce morbidity and, overall, improve trauma provision in East London.

Priority areas

Helmets for All Road Users

A clear priority area for improving road safety for all users would be the extension of fines to cyclists and the emerging group of scooter riders in East London, many of whom do not wear helmets. Research has highlighted the critical importance of helmet use in preventing head injuries [12]. Given the increasing popularity of cycling and scooter use in urban areas, implementing fines could serve as a deterrent, encouraging riders to prioritize their safety and helping to relieve the burden on emergency services.

This issue warrants discussion in Parliament, with consideration given to enacting appropriate legislation.

Funding Positive Initiatives

Endemic violence remains a fundamental issue in East London, driven by a range of socio-economic factors including lack of adult mentorship, poverty, unstable home environments, substance abuse, educational deficiencies, and a pervasive sense of hopelessness [11]. East London, notably Newham, which recorded the highest number of such incidents in 2023 with 642, followed by Tower Hamlets with 547, and Hackney with 546, highlights a significant weakness in trauma provision, particularly regarding prevention efforts [1]. These figures underscore the gravity of the situation and the urgent need for substantial change.

Addressing interpersonal violence requires a multifaceted approach centered on adult mentorship and social inclusion. Establishing mentorship programs could provide at-risk youth with positive role models, guiding them towards promising careers. Furthermore, enhancing engagement with schools through after-school clubs (e.g., sports, skills, and social) and extracurricular activities can foster a sense of belonging [11], enabling young individuals to break out of restrictive social environments, openly discuss their home situations, and consequently address the root causes of aggression. Such initiatives also bridge gaps between different community groups and demographics, promoting greater mutual understanding.

As stated, "Interpersonal violence is driven by social injustice and will be resolved when the communities it affects are empowered to propel change" [11]. In recent years, with the establishment of London's Violence Reduction Unit (VRU) by the Mayor of London [13], a positive shift may be underway. Although the program remains in its early stages, it will be important to monitor its impact closely. The success of such initiatives could reduce pressure on trauma systems. Through clinical governance and data gathering at the Royal London Hospital, the outcomes will be scrutinized, and, it is hoped, positive results will ripple across the London boroughs.

Violence presents a significant challenge to the UK economy, accounting for approximately £2.6 billion of NHS spending annually [14] and contributing to around 2.8% of all ED attendances [15]. These statistics highlight a weakness in the current trauma provision across the United Kingdom.

Funding Prehospital Care

London’s Air Ambulance has significantly improved patient outcomes in East London. However, the fact that the service holds charitable status and is not solely NHS-funded represents a notable weakness and a priority area for improvement. With direct NHS funding, London’s Air Ambulance would have the potential to evolve and expand its services at a faster rate [16].

In 2023, the Helicopter Emergency Medical Service (HEMS) completed 2,007 missions, of which 84 were in East London [16]. Currently, 96% of HEMS funding relies on public support, with the cost of delivering the 24/7 service amounting to approximately £15 million per year. This figure covers fuel costs, operational and charity staff salaries, and medical equipment.

Ongoing public support has enabled the introduction of the Physician Response Unit (PRU) vehicles, with plans underway to introduce a third vehicle to expand coverage to Barking, Havering, and Redbridge, in addition to the existing operations in Tower Hamlets, Newham, and Waltham Forest. The long-term goal of the service is to maintain and develop operations sustainably over the next fifteen years.

Increasing public engagement through greater advertisement in local newspapers, fundraising events, community groups, and school initiatives, as well as the placement of donation pots and collection points, would help secure further funding for the air ambulance charity. Sustained financial support would allow the continuation of vital prehospital care research, ultimately leading to further improvements in trauma provision across London [16].

Rebuild Research Infrastructure

In recent years, efforts to enhance trauma provision in East London have been hindered by the cyberattack on the Trauma Audit Research Network (TARN) [17], which resulted in the loss of historical data. This has significantly affected the ability of healthcare professionals to collect retrospective data necessary for assessing trends and patterns in trauma care. In the absence of such data, primary research has become increasingly difficult.

This incident has highlighted critical vulnerabilities within the current data security infrastructure. Addressing this challenge through the development of a unified, secure, and modernized data system would enhance decision-making and ultimately improve the care provided to patients who have suffered trauma. Large companies commonly use private data security companies to ensure data is protected, and this formula can be emulated by the NHS. 

Moreover, such data must be made accessible to other NHS trusts and, potentially, to healthcare professionals globally. Fostering collaboration through the sharing of insights and innovations, such as those pioneered at Barts Health NHS Trust, could not only strengthen trauma provision in East London but also influence global standards and practice, ultimately benefiting trauma patients worldwide.

## Conclusions

East London has made significant improvements by positioning itself as a national leader through innovation, multidisciplinary collaboration, and community engagement. From pioneering national clinical protocols at the Royal London Hospital to evolving prehospital care and implementing targeted prevention strategies, the region has demonstrated both resilience and responsiveness in the face of complex socio-economic challenges. However, much of this progress has relied on the altruism of healthcare professionals and community members for far too long. Sustainable advancement now requires substantial investment and support.

To ensure lasting impact, trauma care in East London must now transition from relying on goodwill to being underpinned by strategic investment, robust policy frameworks, and secure data infrastructure. This includes fully funding prehospital services like London's Air Ambulance, legislating preventive safety measures, and rebuilding or purchasing reliable data systems to drive informed decision-making. Also, investing in social initiatives that address the root causes of interpersonal violence will be critical to reducing trauma incidence in the long term.

As East London continues to evolve, it offers a compelling model for trauma care that other regions can emulate - one that is not only clinically effective but also socially responsive and future-focused. With coordinated support at local and national levels, the region has the potential to lead a new era of equitable and sustainable trauma provision across the United Kingdom.
